# The Direct Anti-Virulence but Not Bactericidal Activity of Human Neutrophil Elastase against *Moraxella catarrhalis*

**DOI:** 10.3390/ijms24076607

**Published:** 2023-04-01

**Authors:** Justyna Roszkowiak, Siobhán McClean, Aleksandra M. Mirończuk, Daria Augustyniak

**Affiliations:** 1 Department of Pathogen Biology and Immunology, Faculty of Biological Sciences, University of Wroclaw, 51-148 Wroclaw, Poland; 2 School of Biomolecular and Biomedical Sciences and Conway Institute of Biomolecular and Biomedical Research, University College Dublin, Belfield, Dublin 4, D04 V1W8 Dublin, Ireland; 3 Laboratory for Biosustainability, Institute of Environmental Biology, Wroclaw University of Environmental and Life Sciences, 51-631 Wroclaw, Poland

**Keywords:** neutrophil elastase, *Moraxella catarrhalis*, outer membrane vesicles (OMVs), outer membrane proteins (OMPs), complement, anti-virulence action, bactericidal action, inflammation, TEM, OMP CD, COPD

## Abstract

Neutrophil elastase (NE) contributes to innate antibacterial defense at both the intracellular (phagocytosis) and extracellular (degranulation, NETosis) levels. *Moraxella catarrhalis*, a human respiratory pathogen, can exist in an inflammatory milieu which contains NE. No data are available on the action of NE against *M. catarrhalis* or on the counteraction of NE-dependent host defenses by this pathogen. Using time-kill assays we found that bacteria are able to survive and replicate in the presence of NE. Transmission electron microscopy and flow cytometry studies with NE-treated bacteria revealed that while NE admittedly destabilizes the outer membrane leaflet, it does not cause cytoplasmic membrane rupture, suggesting that the enzyme does not target components that are essential for cell integrity. Using LC-MS/MS spectroscopy we determined that NE cleaved at least three virulent surface proteins in outer membrane vesicles (OMVs) of *M. catarrhalis,* including OMP CD, McaP, and TbpA. The cleavage of OMP CD contributes to the significant decrease in resistance to serum complement in the complement-resistant strain Mc6. The cleavage of McaP did not cause any sensitization to erythromycin nor did NE disturb its drug action. Identifying NE as a novel but subtle anti-virulence agent together with its extracellularly not-efficient bactericidal activity against *M. catarrhalis* may facilitate the pathogen’s existence in the airways under inflammation.

## 1. Introduction

Neutrophils are the pivotal cellular components of innate defense that rapidly accumulate at the site of infection. To kill bacterial or fungal pathogens, they use both oxidative and non-oxidative mechanisms during phagocytosis, neutrophil extracellular trap formation (NETosis), and degranulation (exocytosis) of the pre-formed mediators from cytoplasmic granules. Neutrophil elastase (NE) is a serine protease stored in azurophilic granules and is engaged in host defense against Gram-negative but not Gram-positive bacteria [1]. NE plays a multifaceted role in protecting against bacterial infections. The direct antibacterial actions involve general phagosome-dependent and additional non-oxidative bactericidal mechanisms comprising the cleavage of selected outer membrane proteins and as a result membrane destabilization [2,3]. Apart from its antimicrobial proteolytic function, in indirect immunomodulatory mechanism of action, NE promotes cytokines, i.e., TNF-α, MIP-2, and IL-6 expression, which contributes to host anti-*Pseudomonas aeruginosa* defense [4]. Likewise, endogenous elastase at the site of infection/inflammation can process inactive proforms of mammal cathelicidins into active antibacterial peptides [5] as well as synthetic antimicrobial peptides (AMPs) into pharmacologically active peptide D-BMAP18 (a membrane-permeabilizing antimicrobial peptide composed of D-amino acid) with good antibacterial and anti-inflammatory activities [6].

On the other hand, NE may act in strictly opposing way in many chronic disease states, thereby favoring infection. It can cause the impairment of airway epithelial defense against bacteria via degradation of epithelial protein SPLUNC1 (short palate lung and nasal epithelial clone 1) [7]. Alternatively, it may cause tissue damage that decreases host tolerance to lung infection with *Burkholderia* species [8]. The harmful effects of elastase also involve its ability to inactivate the CD14 receptor for lipopolysaccharide, thus reducing the inflammatory response to endotoxin [9] as well as cleaving the phosphatidylserine receptor on macrophages, leading to impairment in clearance of apoptotic cells [10]. To enable cellular migration, elastase can cleave components of the extracellular matrix such as elastin, several types of collagen, or fibronectin [11]. Consequently, released NE may cause serious deleterious effects on surrounding tissues in various chronic diseases, including recurrent aphthous stomatitis, chronic obstructive pulmonary disease (COPD), type 1 diabetes, obesity-related diseases, or chronic kidney disease, regardless of whether it is accompanied by bacterial infections [12,13,14,15]. Furthermore, in chronic lung diseases, NE has also been shown to be associated with induced airway mucus gland hyperplasia, mucus secretion, airway smooth muscle cell proliferation, and airway hyperresponsiveness, features associated with disease progression [16,17,18]. 

As recently documented from a molecular point of view, the essential role in pathological inflammation triggered by *Moraxella catarrhalis* is based on activation the cytosolic innate immune sensor caspase-4/11, gasdermin-D-dependent pyroptosis, and the NLRP3 inflammasome in human and mouse immune cells [19]. *M. catarrhalis* is a Gram-negative human-restricted respiratory pathogen responsible mainly for otitis media in children, and one of the dominant bacteria in exacerbations of COPD or asthma in adults [20,21]. Interestingly, in children suffering from recurrent respiratory tract infections (60%) or asthma (20%), specific anti-*M. catarrhalis* serum IgE has been documented [22]. Furthermore, neonates colonized in the hypopharyngeal region with *M. catarrhalis* and other bacteria such as *S. pneumonia* and *H. influenzae* are at increased risk for recurrent wheeze and asthma early in life [23].

The association between neutrophilic inflammation and a relatively small group of bacterial taxa, which is seen consistently across chronic respiratory conditions, suggests that neutrophilic inflammation also presents a considerable selective pressure on the composition of the airway microbiota [18]. It means that the altered immunity in the inflammatory milieu of the airways may contribute to very specific bacterial colonization and adaptation. Since *M. catarrhalis* is one of the leading epidemiological factors in lung disease exacerbation, it should adapt to function under the aforementioned inflammatory conditions. Indeed, *M. catarrhalis* may contribute to a protease-antiprotease imbalance in part by neutralization of the key antiprotease α 1-antichymotrypsin in the respiratory tract [24]. The duration of asthma with a neutrophilic endotype is also positively correlated with the total relative abundance of *M. catarrhalis* and other pathogens such as *H. influenzae* and *Streptococcus pneumoniae*, which are prevalent within neutrophilic airway secretions [21]. Furthermore, mice infected with *M. catarrhalis* during allergen sensitization had neutrophilic infiltrates in their airways and high levels of proinflammatory cytokines, including IL-6, IL-17, IFN-γ, and TNF-α [25].

Despite the significant role of *M. catarrhalis* in the pathogenesis of chronic respiratory diseases, no study has addressed the role of elastase in immunity against *M. catarrhalis*. Herein, we provide for the first time compelling evidence that neutrophil elastase exerts direct anti-virulence but not bactericidal activity against *M. catarrhalis* in extracellular milieu. The former relies primarily on the degradation of key outer membrane proteins such as OMP CD and McaP, causing the consequent impairment of serum resistance but keeping insensitivity to macrolide antibiotic, respectively. However, despite the anti-virulence action of elastase, the resistance of bacteria to the bactericidal activity of the enzyme, at concentrations exceeding those found in pathological conditions, which are common in COPD exacerbations or bronchiectasis [26,27], may explain their ability to adapt and thrive in its presence.

## 2. Results

### 2.1. Neutrophil elastase at Concentration Representative for Pathological Conditions of Respiratory Tract Does Not Exert Direct Killing against M. catarrhalis

**Figure 1:**
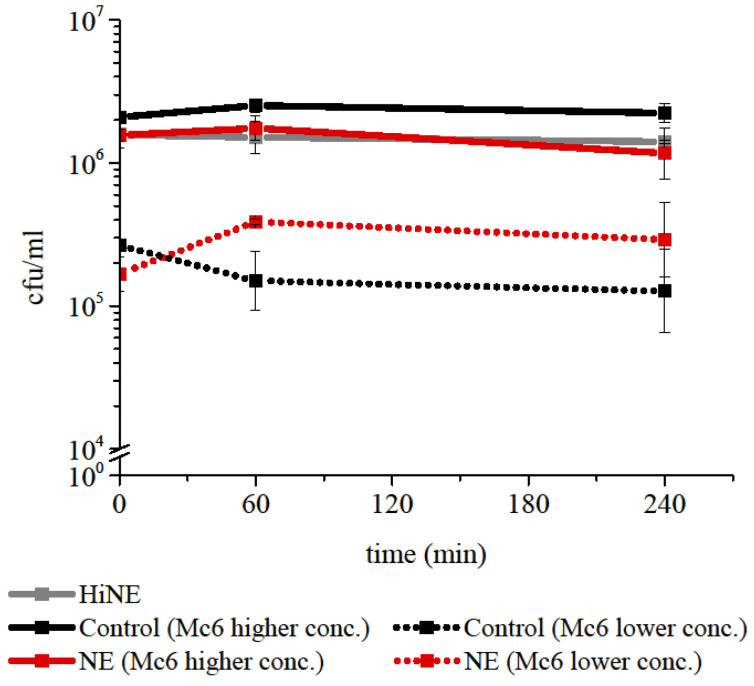
Neutrophil elastase (NE) does not have bacteriolytic activity against *M. catarrhalis*. Bacteria from log phase were incubated for 4 h in the presence of 2 μM of NE and plated in 0, 60, and 240 min. Data are expressed as mean cfu/mL ±  SD from at least two independent experiments performed in triplicate. HiNE—heat inactivated NE (95 °C, 15 min).

**Figure 2:**
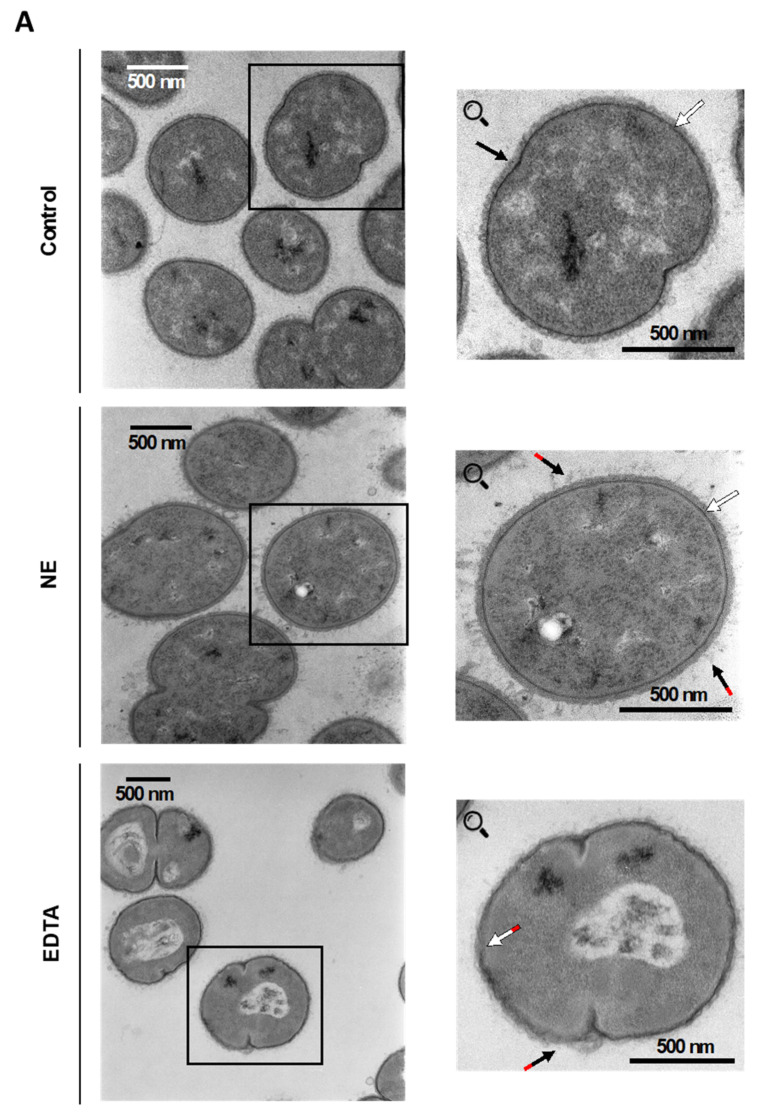

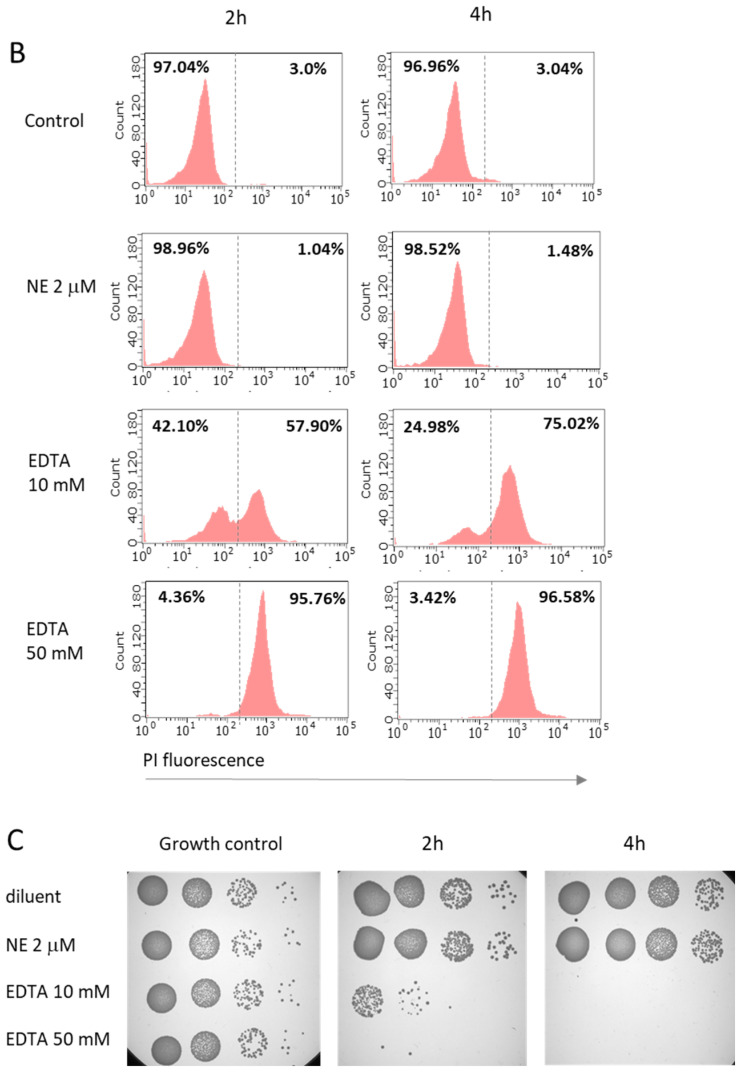
The lack of bactericidal and permeabilizing activity of neutrophil elastase (NE) against *M. catarrhalis Mc6*. (**A**) TEM visualization of bacteria treated with NE. Mc6 were incubated (4 h, 37 °C) with 2 μM NE (middle panel). The negative control was bacteria incubated in diluent only (upper panel), and the positive control was bacteria incubated in the presence of 50 mM EDTA (lower panel). White arrows indicate membranes (outer and inner); black arrows denote lipooligosaccharide. Red arrows indicate changes in disorganization/destabilization of marked structures. TEM images for control and NE, magnification ×22,000; for EDTA, magnification ×16,000. (**B**) Flow cytometry analysis of membrane permeability. Mc6 after 4 h of NE treatment was incubated with 6 μM PI for 15 min at RT and the fluorescence was measured using red fluorescence channel with Guava EasyCyte (Merck). Negative controls were untreated bacteria with PI, and positive controls were bacteria treated with 10 mM and 50 mM EDTA. Representative histograms show the fluorescence intensity of an experiment derived from 3 independent biological replicates. (**C**) Spot plate test visualization of the bacterial growth (dilutions: 10^−1^, 10^−2^, 10^−3^, 10^−4^) following NE and EDTA treatment at time 0, 2 h, and 4 h. Spot tests were performed in parallel with cytometric measurements.

The direct killing activity of NE against Gram-negative bacteria such as *Klebsiella pneumoniae* [1], *Escherichia coli* [2], and *P. aeruginosa* [3] was demonstrated previously. To investigate the bactericidal impact of NE on *M. catarrhalis*, a series of experiments was conducted in vitro using concentrations of NE usually higher than that found in chronic inflammation of the lower respiratory tract. The enzymatic activity of each batch of NE was checked by reaction with a specific fluorogenic substrate MeoSuc-Ala-Ala-Pro-Val-AMC. Assessment of killing ability was performed with or without 2 μM NE using time-kill assays from 0 to 4 h. As illustrated on survival plots, *M. catarrhalis 6* (Mc6) at various cfu/mL was not killed by NE over the 4 h of incubation (Figure 1). The lack of lytic and permeabilization-inducing properties of the enzyme against *M. catarrhalis* was confirmed further using transmission electron microscopy (TEM) observations and flow cytometric measurements. As shown in Figure 2A, TEM images demonstrated some degree of NE-dependent disorganization of lipooligosascharide (LOS) structures, with a characteristic radial morphology and no visible distortion of the inner cell membrane or other deformations of the outer membrane of Mc6 cells. In contrast, the bacteria treated with EDTA (positive control) showed distorted structural integrity of the envelope accompanied by destabilization (disintegration) of both bacterial membranes. The results of undisturbed by NE membrane integrity were confirmed by incubation of bacteria with a dye propidium iodide (PI), that is impermeable to viable cells but intercalates nucleic acids in damaged cells. As shown in Figure 2B,C, for bacteria treated with NE as well as for negative control, the fluorescence intensity was on the similarly very low level (PI-negative cells) and the survival rate was comparable. In contrast, treatment of bacteria with EDTA, which causes an increase in the permeability of cell envelopes, caused cell damage rates, as reflected in both the high percentage of PI-positive cells and the lethal effect seen in the spots. It can be concluded therefore that NE probably does not cleave surface proteins and thus does not facilitate the damage of inner cell membrane of *M. catarrhalis*, which would be considered a lethal effect.

### 2.2. Neutrophil Elastase Degrades Pivotal Outer Membrane Proteins (Virulence Factors) of M. catarrhalis

**Figure 3:**
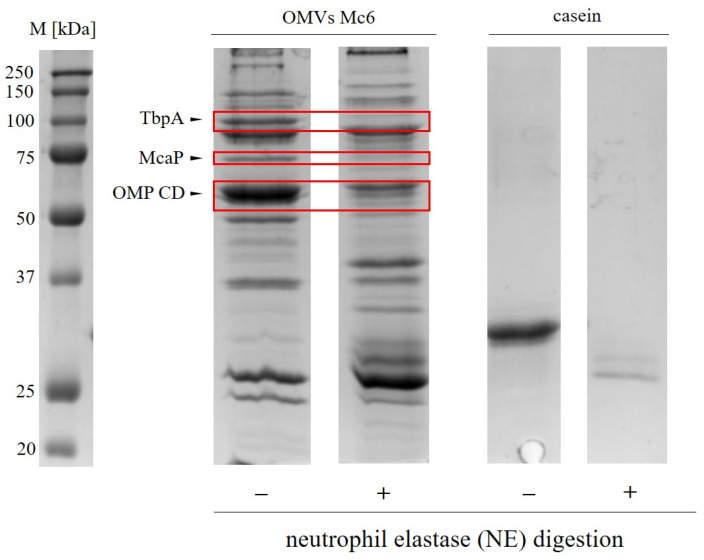
Neutrophil-elastase-mediated proteolysis of *M.catarrhalis* outer membrane proteins. Proteinogram from enzymatic digestion of OMVs Mc6 by human neutrophil elastase (NE). OMVs Mc6 (10 μg) were subjected to enzymatic treatment (NE, 2 μM) for 1 h at 37 °C. As a control OMVs were incubated only in reaction buffer. The degraded by NE outer membrane proteins are indicated by arrows and red frames. NE-dependent digestion of casein (2.5 μg/mL) was performed under analogous conditions (positive control). Proteins were separated of 12% SDS-PAGE electrophoresis of OMVs; the protein profiles were visualized using Coomassie blue staining.

The observed lack of bactericidal activity of NE against Mc6 raises the question of whether, and if so which, surface membrane proteins of Mc6 are susceptible to NE action, and what the other biological consequences are. In addition, given the ability of Mc6 outer membrane vesicles (OMVs) to cause degranulation of PMNs [28], they may contribute to the increased concentration of NE in the immediate vicinity of the bacteria. Treating OMVs with NE showed that at least three key outer membrane proteins (OMPs) of *M. catarrhalis* were cleaved by NE following 1 h incubation at 37 °C (Figure 3). These proteins, corresponding to gel bands of ~50, ~70, ~100, and further analyzed by LC-MS/MS mass spectrometry (Table 1), were identified as transferrin-binding protein TbpA (120 kDa), involved in iron uptake from transferrin [29], and two bacterial adhesins: OMP CD (46 kDa), involved in adhesion and complement resistance [30], and McaP (62 kDa), involved in adhesion and lipolytic activity [31]. These findings indicate that NE exerts a potent proteolytic activity towards three *M. catarrhalis* OMPs which are associated with the virulence of this bacterium. The importance of cleavage of two proteins, namely OMP CD and McaP, were further studied.

Having documented the proteolytic activity of NE against OMP CD and McaP, we posed two research hypotheses. The first hypothesis was that enzymatic degradation of the OMP CD protein, which confers partial complement resistance to human serum, would result in increased bacterial sensitivity to complement. The second hypothesis was that digestion of McaP, a protein displaying esterase activity against macrolide antibiotics [32], would sensitize the bacteria to an exemplary antibiotic of this group, erythromycin. The verification of both hypotheses required demonstrating that the proteolytic activity of NE favors the reduction of resistance of the wild-type *M. catarrhalis* strain to the aforementioned compounds. For these experiments, isogenic mutants of strain Mc6, which were devoid of the OMPs, namely ∆*ompCD* and ∆*mcaP* mutant strains, were used as positive controls (Figure S1).

### 2.3. Degradation of OMP CD by Neutrophil Elastase Sensitizes M. catarrhalis to Complement Action

#### 2.3.1. Complement-Dependent Bactericidal Activity

In preliminary experiments, we confirmed that the complement-resistant wild-type (WT) *M. catarrhalis* Mc6 and its isogenic ∆*ompCD* mutant strain showed different sensitivities to complement in active normal human serum (NHS) while growing comparably in the presence of heat-inactivated NHS (HiNHS,). Specifically, ∆*ompCD* showed a significant decrease in viability in the presence of 25% NHS in comparison to 25% HiNHS. In contrast, the WT strain even grew in the presence of 75% of NHS (Figure S2).

**Figure 4:**
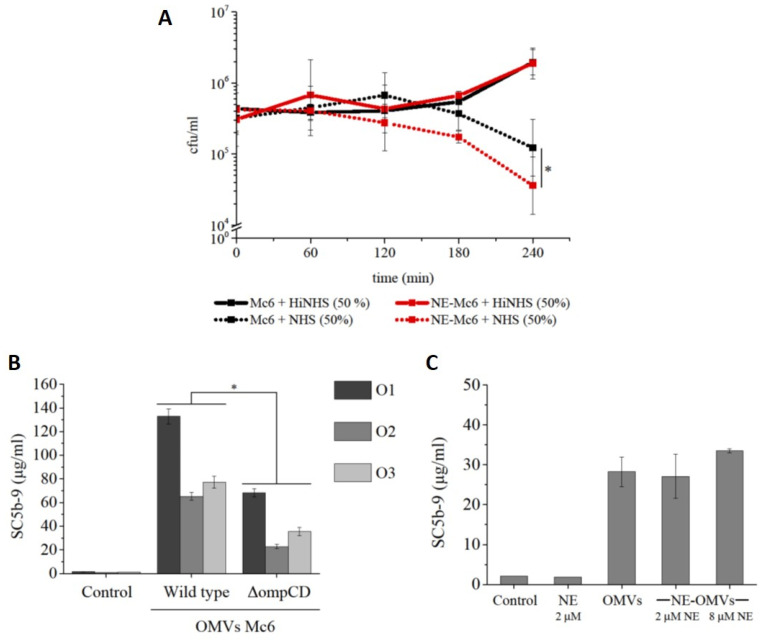
Enzymatic cleavage of surface protein OMP CD by neutrophil elastase (NE) sensitizes *M. catarrhalis* to human complement action. (**A**) Bactericidal activity of human serum complement (NHS) against WT bacteria treated with NE. Bacteria from log phase were enzymatically digested with 2 μM NE (1 h, 37 °C) before 4 h bactericidal assays were performed. Bacteria untreated with NE and incubated in reaction buffer for 1 h at 37 °C were used as controls. Data are expressed as mean cfu/mL ± SD from two independent experiments performed in triplicate. Statistical analysis was performed by Wald–Wolfowitz test (* *p* < 0.05); HiNHS-heat inactivated NHS. (**B**) Human serum complement activation in 90% NHS by OMVs from Mc6 WT and its isogenic mutant following 30 min. incubation at 37 °C as determined by ELISA. Data were analyzed using sera from three volunteers (O1–O3) and are expressed as mean SC5b-9 ± SD from two replicates for each serum. Statistical analysis was performed by T test for independent variables (* *p* < 0.005). (**C**) Activation of human serum complement in 90% NHS by OMVs Mc6 WTe previously cleaved by NE (1 h, 37 °C). Data are expressed as mean SC5b-9 ± SD from two replicates for pooled serum.

To assess the contribution of NE-mediated proteolytic degradation of the surface OMP CD protein to the complement-associated bactericidal actions of NHS, the *M. catarrhalis* WT was incubated for 4 h with 2 μM NE and a subsequent additional 2 h incubation was performed with either NHS or HiNHS. As shown in Figure 4A, the WT strain subjected to proteolytic degradation by NE became significantly more susceptible to complement action by NHS in comparison to the intact bacteria. At the same time, no reduction in the survival of enzymatically digested Mc6 was observed in the HiNHS control serum. These finding indicates that NE is able to degrade OMP CD in the outer membrane of intact *M. catarrhalis*, resulting in its greater sensitivity to NHS complement.

#### 2.3.2. Activation of Terminal Complement SC5b-9 Component

Next, knowing that NE can degrade OMP CD, we decided to verify the potential of NE-treated OMPs to activate the complement system and that the proteolytic action of NE did not interfere with complement activity. Using an ELISA assay, the quantity of the soluble terminal membrane attack complex SC5b-9 was measured as an indicator of complement activation. As shown in Figure 4B, the OMP CD protein-rich OMVs from *M. catarrhalis* WT strain were potent activators of complement cascade. However, although the absence of OMP CD protein in OMVs from the ∆*ompCD* mutant strain significantly attenuated SC5b-9 formation (Figure 4B), the enzymatic digestion of OMVs with clinically-relevant concentrations of NE did not affect the activation of complement compared with non-digested OMVs (Figure 4C). This finding shows that NE treatment of OMVs does not affect complement activation.

### 2.4. Degradation of McaP by Neutrophil Elastase Does Not Sensitize M. catarrhalis to Erythromycin Action

McaP, a conserved autotransporter has adhesive properties and mediates adherence to human epithelial cells [31,32]. This protein also displays esterase activity, [32], which determines one of the mechanisms of macrolide resistance [33].

**Figure 5:**
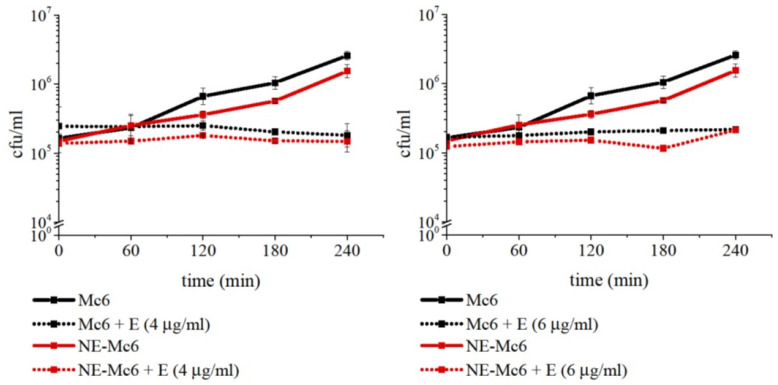
Enzymatic cleavage of surface protein McaP by neutrophil elastase (NE) does not sensitize *M. catarrhalis* to erythromycin. The curves on activity of erythromycin against WT log phase bacteria pre-treated (1 h, 37 °C) with 2 μM NE and then incubated for 4 h with antibiotic are shown. Data are expressed as mean cfu/mL ± SD from two independent experiments performed in triplicate.

Initially, examining the sensitivity of Mc6 WT to erythromycin (macrolide antibiotic), we showed, that for ~2.5–5 × 10^5^ cfu/mL, the minimum inhibitory concentration (MIC) and the minimum bactericidal concentration (MBC) were, respectively, 0.125 µg/mL and 0.5 µg/mL. As expected, the isogenic Δ*mcaP* Mc6 mutant strain was significantly more sensitive to this antibiotic. In time-kill assays, the lethal effect of supra-MICs concentration of erythromycin occurred 1 h post-incubation. Applying the same concentrations of antibiotic to the WT strain only produced a bacteriostatic effect (Figure S3). The results obtained confirmed the contribution of the McaP protein to the survival of Mc6 bacteria in the presence of erythromycin. In the next step, erythromycin-dependent bactericidal tests were performed on Mc6 WT bacteria that had been previously treated with NE. In this case, despite the fact that NE digests the McaP outer membrane protein of the *M. catarrhalis* Mc6 (Figure 3), no sensitization of this pre-exposed Mc6 bacterium to erythromycin was observed, despite extended incubation times (Figure 5). Nevertheless, besides the lack of enhanced erythromycin activity after NE treatment, it is worth adding that the presence of NE in the environment does not disturb its bacteriostatic drug action.

### 2.5. M. catarrhalis Is a Potent Inducer of Neutrophil Elastase Release

**Figure 6:**
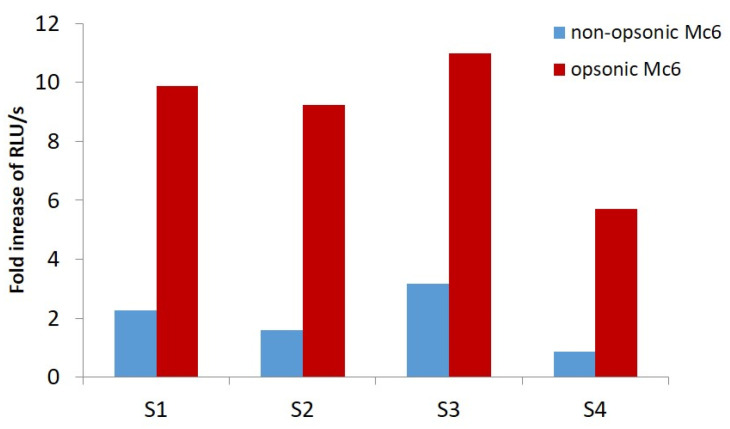
*M. catarrhalis* Mc6 is effective stimulator of human PMNs degranulation and elastase release. Human neutrophil samples at ~1 × 10^7^ cell/mL from four volunteers S1–S4 were primed with cytochalasin D (5 μg/mL), incubated for 30 min with bacteria preincubated with 10% pooled human sera (opsonic) or not (non-opsonic), and assayed for the release of elastase. Activity of enzyme was determined using fluorogenic substrate, MeoSuc-Ala-Ala-Pro-Val-AMC, and the increase in fluorescence was measured using Varioskan™ Flash Multimode Reader, Thermo Scientific, Vantaa, Finland) at Ex. *λ* = 370  nm and Em. *λ* = 445 nm. Data are expressed as fold increase of RLU/s in comparison to non-stimulated control for each individual supernatant after degranulation.

Given the ability of *M. catarrhalis* to cause degranulation of PMNs, they may contribute to the increased concentration of this proteolytic enzyme in the immediate vicinity of the bacteria. Previously, we have shown that OMVs released by *M. catarrhalis* are potent inducers of NE release from PMNs neutrophils [28]. Here, we documented the differences in magnitude of NE release due to PMN degranulation in response to bacteria either opsonized or not by human serum (opsonic versus non-opsonic manner). As shown in Figure 6, in opsonic conditions, NE release was 5.7- to 11-fold higher in comparison to the unstimulated control, depending on the blood donor. In non-opsonic conditions, this increase was noticeably lower, from 1.2 to maximally 3.3-fold. These results indicated that the opsonized Mc6 induced the mean ~4-fold ± 0.52 SD higher increase in elastase release comparing to non-opsonized bacteria. By inducing the release of NE under a variety of immune conditions, *M. catarrhalis* contributes to enhancing the inflammatory environment, which facilitates its persisting.

## 3. Discussion

Excessive neutrophilic inflammation accompanied by the impaired function of neutrophils is a hallmark of lower respiratory tract infections and chronic pulmonary diseases, including acute respiratory distress syndrome COPD or neutrophilic asthma [14,18,34]. For example, in COPD, patients’ neutrophils are described as aberrant due to abnormal degranulation, phagocytosis, high ROS generation, and NET formation [35]. Furthermore, it was shown that NETs are more abundant in sputum from patients with severe COPD and are associated with more frequent exacerbations as well as loss of microbiota diversity and *Haemophilus* species dysbiosis [36]. There is increasing evidence that an overwhelming NET response correlates with poor outcome also in other lung-related diseases such as bacterial pneumonia, cystic fibrosis, or influenza. The detrimental effects that NETosis causes, such as destruction of epithelial and endothelial cells, vessel occlusion, or additional neutrophil recruitment and activation [37] are partly responsible for this. Likewise, neutrophils from patients with neutrophilic asthma display enhanced migration but diminished phagocytic efficiency compared with healthy controls [38].

In the case of bacterial exacerbation of inflammatory disorders, bacteria are exposed to granule contents containing elastase during neutrophil degranulation or NET-osis. As was documented in this paper, the exposure of *M. catarrhalis* to NE at 2 μM (60 μg/mL) did not result in the loss of their cocci-like morphology, inner-membrane damage or increased permeability. For other Gram-negative bacteria, using the same or even lower concentrations of the enzyme, a nonoxidative mechanism of bactericidal action of NE involving degradation of pivotal OMPs that facilitate osmotic lysis has been proposed for OmpA *E. coli* [2] and OprF *P. aeruginosa* [3]. Our observed lack of bacterial death in *M. catarrhalis* after exposure to NE used in concentrations exceeding those documented for lower airways in bronchoalveolar lavage fluid (BALF) in COPD exacerbations or bronchiectasis [26,27] indicates that, in these conditions, potential degradation of any of the highly expressed surface proteins by NE is not sufficient to either destroy cell wall integrity or to locally attenuate wall thickness, which would facilitate osmotic lysis. The lack of, or reduced sensitivity to, NE may be another strategy that allows the bacterium to survive extracellularly in its presence, although we cannot exclude the possibility that bactericidal activity could be observed in phagolysosomal compartments inside neutrophils, where the concentration of this enzyme should be much higher. To date, other defense strategies of *M. catarrhalis*, which allow the bacteria to overcome the inflammatory conditions, have also been documented. For example, *M. catarrhalis* evaded neutrophil oxidative stress responses via induction of less ROS and reduced NETosis in differentiated HL-60 neutrophils [39].

Although we did not observe a direct elastase-dependent bacteriolytic effect or inner-membrane perturbation using 2 μM of enzyme, we have shown for the first time that neutrophilic elastase caused the proteolytic degradation of three important outer-membrane proteins of this bacterium, i.e., OMP CD, McaP, and TbpA, as was determined by LC-MS/MS analyses. The functional significance of this phenomenon for two of the proteins mentioned, namely OMP CD and McaP, has been further explored. OMP CD is a highly conserved and abundantly expressed *M. catarrhalis* surface protein identified as a target of serum IgG antibodies to surface epitopes in the majority of adults with COPD who cleared this pathogen as well as mucosal IgA in COPD patients [40,41]. OMP CD is recognized intensively by cross-reactive intraspecies antibodies from mice sera and human sera in healthy children and those with otitis media [42,43]. Functionally, this protein is involved in complement resistance [32]. Initially, using a constructed isogenic ∆ompCD mutant of Mc6 defective in expression of OMP CD as an internal control, we confirmed the involvement of this surface protein in complement resistance by showing that bacteria lacking OMP CD die in the presence of the complement cascade in contrast to the wild-type strain. Interestingly, although the absence of OMP CD protein in OMVs significantly attenuates the activation of the terminal SC5b-9 complement complex, enzymatic digestion of OMVs with clinically relevant concentrations of NE (2 µM) does not inhibit the formation of the aforementioned complex as compared to OMVs that are not digested by NE. These results imply that the proteolytic activity of NE against OMVs did not interfere with complement activation. The finding that NE-dependent proteolytic degradation of OMP CD sensitizes complement-resistant *M. catarrhalis* Mc6 to bactericidal action of complement, contributing to the significant decrease of this resistance, is important and a new observation. It indicates that NE exerts direct anti-virulence activity against *M. catarrhalis*, making it more susceptible to the action of this humoral innate mechanism.

Unlike many Gram-negative bacteria which are resistant to macrolide antibiotics, most strains of *M. catarrhalis* are sensitive to these hydrophobic compounds, including erythromycin [44]. However, the presence of OMPs with esterase activity may potentially contribute to macrolide resistance. It has been previously demonstrated that a lack of McaP expression abolishes the esterase activity of isogenic *M. catarrhalis* O35E mutant and considerably decreases its adherence to several human cell lines [32]. The esterase activity of McaP against erythromycin should result in its degradation. In the absence of McaP, this bactericidal activity of the antibiotic is expected to be enabled. However, when analyzing the consequences of NE-dependent digestion of McaP, we did not show any greater sensitization to erythromycin beyond inhibition of bacterial growth in its presence, as we observed for bacteria incubated in the presence of antibiotic alone. The observed lack of bactericidal effect of erythromycin in the presence of surface McaP partially digested by elastase can be explained by the sufficiently high expression level of McaP protein on bacterial cells, thereby retaining esterase activity against erythromycin despite the action of NE. Alternatively, the proteolytic action of NE did not cleave the site of McaP responsible for the esterase activity. Other OMPs encoding genes, including *uspA2* and *uspA2H,* also are reported to be engaged in macrolide resistance in *M. catarrhalis*. Furthermore, macrolide-resistant isolates exhibited enhanced adhesion when compared with macrolide-susceptible isolates, indicating they were more pathogenic [45].

Overall, despite the fact that our studies did not reveal any direct bactericidal action of NE against *M. catarrhalis*, we showed a new beneficial indirect role for this enzyme in the innate immune response against this bacterium. It involves the decrease of the resistance of *M. catarrhalis* to human serum complement.

Bacteria and their OMVs can induce neutrophil granule exocytosis [28,46]. Moreover, bacterial pathogens can manipulate neutrophil degranulation and by inhibiting, dysregulating, or inducing excessive neutrophil degranulation, bacteria can skew the protective effects of neutrophil degranulation in a way that ultimately benefits the pathogen and worsens disease [47]. This virulence strategy is used by *Shigella flexnerii*, which utilizes antimicrobial proteins released by degranulation to increase adhesion efficiency followed by hyperinvasion into epithelial cells [48]. Since the OMVs released can disseminate over significant distances, OMV-dependent degranulation of PMNs may be another virulence mechanism that triggers cellular exocytosis away from the bacteria. This could both delay the direct contact between the pathogen and PMNs and disarm PMNs by protecting bacteria from the anti-virulent effects of elastase. The protective role of vesicles against the deleterious effects of released neutrophil granule components has so far been demonstrated for several Gram-negative pathogens. For example, *Porphyromonas gingivalis* deploys OMVs decorated with gingipains for a neutrophil-deceptive strategy to degrade released external MPO and LL-37, creating a favorable inflammatory niche as well as avoiding killing [49]. We have previously demonstrated that neutrophils stimulated by *M. catarrhalis* OMVs released both azurophilic and secondary granules and that these OMVs caused cell death of respiratory epithelial cells [28]. In the present work, we found that antibody-opsonized bacteria induced significantly stronger NE release than non-opsonized bacteria, suggesting that under a variety of immune conditions *M. catarrhalis* contributes to enhancing the inflammatory niche in which it can persist.

Importantly, the level of free elastase in the lungs of severe COPD patients is significantly higher than in healthy individuals [50]. This enzyme is recognized also as a valuable biomarker for distinguishing the bacterial exacerbation in patients in COPD [27]. Thus, the bacteria- or OMV-dependent elastase release, trapping, and finally utilization, together with PMN depletion and exhaustion, may facilitate the adaptation of *M. catarrhalis* in countering lower respiratory tract defense.

In conclusion, the ability of *M. catarrhalis* to provoke neutrophil elastase release which does not seem to be effective as a bactericidal agent against these bacteria, at least in extracellular inflammatory milieu, as well as the identification and characterization of novel NE proteolytic targets within *M. catarrhalis* OMPs, broaden our understanding of how these bacteria contribute to enhancing the inflammation in which they persist and counteract host defense mechanisms.

## 4. Materials and Methods

### 4.1. Reagents

The following reagents were used: acetonitrile (Sigma-Aldrich, Saint Louis, MO, USA); BHI or BHI agar (Brain Heart Infusion, OXOID, Basingstoke, UK); Columbia agar with 5% sheep blood (BioMérieux, Warszawa, Poland); Bradford reagent (Protein Assay Dye Reagent Concentrate, Bio-Rad, München, Germany); GelCode blue stain reagent (Thermo Scientific, Rockford, IL, USA); human neutrophil elastase and elastase fluorogenic substrate MeoSuc-Ala-Ala-Pro-Val-AMC (Calbiochem, San Diego, CA, USA); Hank’s Buffer with Ca^2+^, Mg^2+^ (HBSS, Lonza, Walkersville, MD, USA); kanamycin and erythromycin (ROTH, Karlsruhe, Germany); Percoll (GE Healthcare, Uppsala, Sweden); dextran (Sigma, St. Louis, MO, USA); PBS (IITD PAN, Wroclaw, Poland); propidium iodide (Invitrogen by Thermo Fisher Scientific, Eugene, OR, USA); Trypan blue (ICN Biomedicals, Eschwege, Germany); sequencing grade modified porcine trypsin (Promega, Madison, WI, USA); Zwittergent 3-14 (Calbiochem, Darmstadt, Germany).

### 4.2. Microbial Strains and Growth Condition

*M. catarrhalis* Mc6, described previously [43], and its study isogenic mutants generated in this study were used. WT strain was grown on Columbia agar with 5% sheep blood, BHI agar plates, or BHI broth. Mutants were grown on BHI supplemented with 20 μg/mL of kanamycin. Strains were cultivated at 37 °C.

### 4.3. PMNs Isolation

Polymorphonuclear (PMNs) cell fraction enriched in neutrophils was isolated as describe previously [28]. Briefly, heparinized blood from healthy volunteers, aged 20–45 years, was mixed in a 1:1 ratio with 2% dextran *w*/*v* dextran in PBS buffer, pH 7.4, and incubated for 30–40 min at RT for erythrocytes sedimentation. The 3–6 mL of PMN-rich plasma collected was carefully transferred to a discontinuous Percoll gradient (61% and 76% in 0.9% NaCl) and centrifuged (320× *g*/10 min, RT). After centrifugation, the PMNs fraction between both Percoll layers was collected in a sterile falcon tube and washed twice by centrifugation (320× *g*/10 min, RT) with erythrocyte lysis buffer (150 mM NH4Cl; 10 mM KHCO3; 0.3 mM EDTA; pH 7.4). Finally, the cells were resuspended in HBSS. Isolated neutrophils were assessed for viability with the trypan blue exclusion assay.

### 4.4. PMNs Degranulation Assay

PMNs were subjected to degranulation as previously described [28]. Briefly, 1 × 10^7^ cells/mL were gently mixed and 500 μL was aliquoted into polystyrene cell culture tubes. To obtain degranulation, all samples (without spontaneous degranulation samples) were primed with cytochalasin D (5 μg/mL) for 5 min at 37 °C, 5% CO_2_, followed by a further 30 min incubation (37 °C, 5% CO_2_) with stimulants including opsonized (10% pooled heat inactivated (56 °C, 30 min) human serum) or serum non-opsonized *M. catarrhalis* Mc6 at ~5 × 10^8^ and 1 µM fMLP as positive control. Subsequently, samples were centrifuged (320 × g/10 min, RT), and after subsequent centrifugation of supernatant, the bacteria-free supernatants were aliquoted and stored at −20 °C. 

### 4.5. Neutrophil Elastase Activity Measurement

Neutrophil elastase (NE) activity was determined by measuring the cleavage of the fluorogenic NE substrate, MeoSuc-Ala-Ala-Pro-Val-AMC, dissolved in Hank’s Balanced Salt Solution (HBSS) reaction buffer at pH 7.5, containing 0.1% (*w*/*v*) HEPES, 10% (*v*/*v*) DMSO, and 150 mM NaCl, as determined previously [28,51]. The working substrate concentration that gave the linear relationship (increase in fluorescence) was 100 μM as determined in preliminary calibration curve experiments with various concentrations of elastase. Cell-free suparnatants after degranulation were added to the substrate in a 1 : 1 ratio in a volume of 50 μL each and were immediately measured using the 96-well flat-bottom black microplate (NUNC). The cleavage rate of the substrate measured for 30 min at 37 °C as the increase in fluorescence was monitored spectrofluorometrically (Varioskan™ Flash Multimode Reader, Thermo Scientific) at excitation wave *λ* = 370 nm and emission *λ* = 445 nm.

### 4.6. Flow Cytometry Analysis

To measure the activity of NE in permeabilization of bacterial membranes, the method described previously was used [52]. Briefly, 18 h Mc6 was recultivated in BHI until early-log phase (OD_600_ = 0.25). The bacteria were washed with PBS (pH 7.4), resuspended in PBS-1% BHI (*w*/*v*) and diluted to ~2–4 × 10^6^ cfu/mL. The cells (100 μL) supplemented with NE, EDTA (positive control), or buffer (negative control) were incubated at 37 °C up to 4 h (thermoblock) and then treated with 6 μM PI for 15 min at room temperature. The samples were suspended in 250 μL PBS, diluted at least 10× in PBS and analyzed with GUAVA EasyCyte flow cytometer (Merck) by measurement of 5000 events on red fluorescence channel. Data were analyzed using GUAVA EasyCyte software (guavaSoft 3.3) Tests were performed in 3 independent biological replicates.

### 4.7. In Gel Trypsin Digestion and Peptide Identification by LC MS/MS Analysis

Gels were rinsed with HPLC-grade water. Excised bands were destained in 100 µL of 100 mM ammonium bicarbonate/acetonitrile solution for 30 minutes at room temperature and then washed with 500 µL of neat acetonitrile. Gel pieces were then covered with 10 ng/µL porcine trypsin solution in 10 mM ammonium bicarbonate/10% (*v*/*v*) acetonitrile and incubated for 2 h on ice followed by overnight incubation at 37 °C. After digestion, samples were centrifuged and supernatant aliquots were withdrawn and stored at −20 °C until LC MS/MS analysis. The analyses were performed on an Ion Trap LC/MS/MS spectrometer (Agilent Technologies, Santa Clara, CA USA). The resulting peptide mass fingerprints and LC MS/MS fragmentation spectra were identified using the MASCOT (http://www.matrixscience.com) and BLAST engines [53] searching *M. catarrhalis* protein databases.

### 4.8. Time-Kill Assay for WT and Isogenic Mutants

For the time kill assay from 0 to 4 h, overnight cultures of Mc6 WT, ∆*ompCD*, or ∆*mcaP* were recultivated until early log-phase (OD_600_ = 0.25–0.3) in relevant media. The bacteria were diluted in 1% (**w*/*v**) BHI-PBS to obtain ~2–4 × 10^6^ cfu/mL and incubated in the presence of NE, NHS, HiNHS, or erythromycin in a final volume of 100 μL for 0, 1, and 4 h at 37 °C. At each time point suspensions were 10-fold serially diluted with and 10 μL aliquots were plated in triplicate on BHI agar plates or alternatively in spots. The plates were incubated overnight at 37 °C, and cfu/mL were calculated.

### 4.9. Bactericidal Activity of Serum Complement or Erythromycin against NE-Treated Bacteria

For enzymatic digestion, the early-log phase bacteria at ~5 × 10^5^ cfu/mL were mixed with 2 µM NE in an elastase HBSS buffer containing 0.1% HEPES, 10% DMSO, 150 mM NaCl, 1% BHI (pH 7.5) in a final volume of 100 µL. The not enzymatically treated bacteria were used as control samples. Bacteria were incubated for 4 h at 37 °C in water bath.

Next. the NE-treated as well as NE-non-treated bacteria were divided into equal volumes and used in bactericidal tests with 25% or 50% normal human serum (NHS) as well as 4 or 6 μg/mL of erythromycin (E). Simultaneously, the incubation of bacteria in the presence of heat-inactivated serum (HiNHS) and appropriate diluent was included as negative and positive controls, respectively. To assess the bactericidal effect at 0, 60, 120, and 240 min of the experiment, 10 μL each of bacterial suspensions incubated in a water bath at 37 °C were 10-fold serially diluted and then 10 μL aliquots were plated in triplicate on BHI agar plates. The plates were incubated overnight at 37 °C. The colony counts and cfu/mL were calculated next day.

### 4.10. Complement Complex SC5b-9 Activation

Briefly, 10 µL of OMVs in veronal buffer (pH 7.4) to obtain final vesicle protein concentrations of 20 µg/mL were added to 90 µL of NHS. The negative control was NHS with the addition of 10 µL of veronal buffer. The samples were incubated for 30 min at 37 °C, diluted in the range 100–2000×, and concentration of soluble SC5b-9 was determined by ELISA kit (MicroVue SC5b-9 Plus, Quidel; Athens, OH, USA) according to the manufacturer’s instructions. The absorbance at λ = 450 nm was read using Varioskan™ LUX multimode microplate reader (Thermo Scientifc, Vantaa, Finland). 

### 4.11. Outer Membrane Vesicles Isolation

Outer membrane vesicle (OMV) isolation was performed as we reported previously [43]. Briefly, the 18 h pre-culture of *M. catarrhalis 6* (Mc6) was diluted 50 × in 500 mL brain–heart infusion (BHI) media and incubated at 37 °C for 16–18 h with orbital shaking (150 rpm). The culture was centrifuged at 8000 rpm for 15 min at 4 °C. The supernatant was collected and passed through 0,22 µm pore size filter vacuum pump (Merck, Millipore). The filtrate was concentrated using 50 kDa vivaspin centrifugal concentrators (Amicon ultra, Merck Millipore, Cork, Ireland) at 5000× *g* for 30 min at 4 °C. The concentrated supernatant was thereafter ultracentrifuged overnight (100,000× *g*, at 4 °C) using Beckman Coulter Optima ultracentrifuge (model L-90K, Palo Alto, CA, USA). The pellet containing OMVs was re-suspend in 500 µL of sterile PBS buffer (pH 7.4), aliquoted, and stored in −20 °C. The sterility of OMVs was confirmed on BHI agar. The protein concentration in OMV preparation was measured using Qubit fluorometer (Life Technologies Corporation, Carlsbad, CA, USA), and the quality of OMVs preparation was confirmed in 12% SDS-PAGE stained with GelCode blue stain reagent.

### 4.12. Outer Membrane Protein Isolation

Outer membrane proteins (OMPs) were isolated with zwitterionic detergent Zwittergent 3–14 according to our method described in [42]. Briefly, the bacteria from 200 mL of culture were suspended in 5 mL of 1 M sodium acetate buffer containing 1 mM β-mercaptoethanol, pH 4.0). To this suspension, a 45 mL volume of a solution of 0.5 M CaCl_2_ containing 5% Zwittergent was added and stirred for 1 h at room temperature. The nucleic acids were precipitated by adding 12.5 mL of cold absolute ethanol and subsequently centrifuging the solution (17,000× g, 10 min., 4 °C). The pellet was discarded and the proteins remaining in the supernatant were precipitated by adding 187 mL of cold ethanol and collected by centrifugation (17,000× *g*, 20 min., 4 °C). The pellet was air dried and then resuspended in 10 mL of Z buffer (0.05% Zwittergent, 50 mM Tris, 10 mM EDTA; pH 8.0). This mixture was stirred for 1 h at room temperature and centrifuged at 12,000× g for 10 min. at 4 °C, and the soluble fraction containing OMPs was retained. The OMPs were divided into aliquots and stored at −80 °C. The quantity and quality of OMPs preparation was confirmed using Bradford reagent and 12% SDS-PAGE stained with GelCode blue stain reagent, respectively.

### 4.13. TEM

Briefly, 18 h cell culture of *M. catarrhalis* in BHI was centrifuged and rinsed in PBS. The pellet was fixed in 1 mL of cacodylate buffer (0.2 M sodium cacodylate, 0.2 M HCl, pH 7.4) supplemented with 2.5% glutaraldehyde and incubated 8–10 h at room temperature (RT). The suspension was rinsed by centrifugation (3000× *g*, 10 min., RT) several times with cacodylate buffer. The resultant pellet was postfixed in cacodylate buffer containing 1% OsO_4_ for 2 h at RT and rinsed. The samples were subsequently dehydrated in a series of ethanol concentrations and embedded in Epon 812. Thin sections were cut with an ultramicrotome (Reichert-Jung) equipped with a diamond knife and stained with 2% uranyl acetate and lead citrate. The samples were then visualized with a TEM (TESLA BS 540, Brno, Czech Republic) operated at 80 kV.

### 4.14. Construction of Mc6 Isogenic Mutants

To construct *M. catarrhalis* 6 ∆*ompCD* mutant, the plasmid pGEM-∆*ompCD* carrying deletion cassette was transformed into *M. catarrhalis* Mc6 strain according to procedure described before [54]. Kanamycin-resistant transformants were selected and the proper deletion was verified by the primers ompCD-F2 (5′-CATATGGGTGTGACAGTCAGCCCACTAC-3′) and ompCD-R2 (5′- GGATCCCTGGCGATATGCCCGAACTG-3′). For further studies, the clone no. 2 was selected.

To obtain *M. catarrhalis* 6 ∆*mcaP* mutant, WT Mc6 was transformed with plasmid pJTmcaPnpKAN, as described before [32]. Kanamycin-resistant transformants were selected and the proper deletion was verified by the primers P1 (5′-CGCAATAAAGATCACCATGCTTG-3′) and P2 (5′-CGGGATCCCGCTGACACATTGCATTGATAAA-3′). For further studies, the clone no. 3 was selected.

### 4.15. Statistical Analysis

The data were expressed as the mean ± SD and analyzed for the significant difference using the Statistica (version 13.3) software (StatSoft, Krakow, Poland). Differences were considered statistically significant if *p* < 0.05.

## Figures and Tables

**Table 1:** OMPs proteolytically cleaved by NE as determined by LC-MS/MS spectrometry.

Protein Name and Description	MW ^1^ [kDa]	Sequence Coverage (%)	Score ^2^	No. of Unique Peptides
TbpA (Transferrin binding protein A)	119.3	61.45	163.57	53
McaP (*Moraxella catarrhalis* adherence protein)	71.3	70.15	71.45	28
OMP CD (Outer membrane protein CD)	48.3	56.95	236.58	29

Proteins determined by LC-MS/MS of excised 1DE gel bands. ^1^ Theoretical molecular mass was determined by Mascot. ^2^ Score ranges represent MS/MS ion scores determined by peptide mass fingerprinting. Scores deemed to be significant by Mascot (*p* < 0.05) are shown.

## Data Availability

The data presented in this study are available on request from the corresponding author. The genome sequences of Mc6 can be found in GenBank under accession number CP010901.

## Supplementary Materials

The following supporting information can be downloaded at: https://www.mdpi.com/article/10.3390/ijms24076607/s1.

## References

[1] Belaaouaj A., McCarthy R., Baumann M., Gao Z., Ley T.J., Abraham S.N., Shapiro S.D. (1998). Mice lacking neutrophil elastase reveal impaired host defense against gram negative bacterial sepsis. Nat. Med..

[2] Belaaouaj A., Kim K.S., Shapiro S.D. (2000). Degradation of outer membrane protein a in *Escherichia coli* killing by neutrophil elastase. Science.

[3] Hirche T.O., Benabid R., Deslee G., Gangloff S., Achilefu S., Guenounou M., Lebargy F., Hancock R.E., Belaaouaj A. (2008). Neutrophil elastase mediates innate host protection against *Pseudomonas aeruginosa*. J. Immunol..

[4] Benabid R., Wartelle J., Malleret L., Guyot N., Gangloff S., Lebargy F., Belaaouaj A. (2012). Neutrophil elastase modulates cytokine expression: Contribution to host defense against *Pseudomonas aeruginosa*-induced pneumonia. J. Biol. Chem..

[5] Scocchi M., Skerlavaj B., Romeo D., Gennaro R. (1992). Proteolytic cleavage by neutrophil elastase converts inactive storage proforms to antibacterial bactenecins. Eur. J. Biochem..

[6] Degasperi M., Sgarra R., Mardirossian M., Pacor S., Maschio M., Scocchi M. (2022). Elastase-activated antimicrobial peptide for a safer pulmonary treatment of cystic fibrosis infections. Antibiotics.

[7] Jiang D., Wenzel S.E., Wu Q., Bowler R.P., Schnell C., Chu H.W. (2013). Human neutrophil elastase degrades SPLUNC1 and impairs airway epithelial defense against bacteria. PLoS ONE.

[8] Sahoo M., Del Barrio L., Miller M.A., Re F., Weiss D. (2014). Neutrophil elastase causes tissue damage that decreases host tolerance to lung infection with *Burkholderia* species. PLoS Pathog..

[9] Le-Barillec K., Si-Tahar M., Balloy V., Chignard M. (1999). Proteolysis of monocyte CD14 by human leukocyte elastase inhibits lipopolysaccharide-mediated cell activation. J. Clin. Investig..

[10] William Vandivier R., Fadok V.A., Hoffmann P.R., Bratton D.L., Penvari C., Brown K.K., Brain J.D., Accurso F.J., Henson P.M. (2002). Elastase-mediated phosphatidylserine receptor cleavage impairs apoptotic cell clearance in cystic fibrosis and bronchiectasis. J. Clin. Investig..

[11] Chua F., Laurent G.J. (2006). Neutrophil elastase: Mediator of extracellular matrix destruction and accumulation. Proc. Am. Thorac. Soc..

[12] Polańska B., Niemczuk M., Augustyniak D., Jankowski A. (2006). Plasma neutrophil elastase in children with recurrent aphthous stomatitis. Cent. Eur. J. Immunol..

[13] Polańska B., Augustyniak D., Makulska I., Niemczuk M., Zwolińska D., Jankowski A. (2010). Elastase, α1-proteinase inhibitor, and interleukin-8 in pre-dialyzed and hemodialyzed patients with chronic kidney disease. Pediatr. Int..

[14] Jasper A.E., Mciver W.J., Sapey E., Walton G.M. (2019). Understanding the role of neutrophils in chronic inflammatory airway disease. F1000Research.

[15] Herrero-Cervera A., Soehnlein O., Kenne E. (2022). Neutrophils in chronic inflammatory diseases. Cell. Mol. Immunol..

[16] Nadel J.A. (2000). Role of neutrophil elastase in hypersecretion during copd exacerbations, and proposed therapies. Chest.

[17] Koga H., Miyahara N., Fuchimoto Y., Ikeda G., Waseda K., Ono K., Tanimoto Y., Kataoka M., Gelfand E.W., Tanimoto M. (2013). Inhibition of neutrophil elastase attenuates airway hyperresponsiveness and inflammation in a mouse model of secondary allergen challenge: Neutrophil elastase inhibition attenuates allergic airway responses. Respir. Res..

[18] Crisford H., Sapey E., Rogers G.B., Taylor S., Nagakumar P., Lokwani R., Simpson J.L. (2021). Neutrophils in asthma: The good, the bad and the bacteria. Thorax.

[19] Tuipulotu D.E., Feng S., Pandey A., Zhao A., Ngo C., Mathur A., Lee J., Shen C., Fox D., Xue Y. (2023). Immunity against *Moraxella catarrhalis* requires guanylate-binding proteins and caspase-11-NLRP3 inflammasomes. EMBO J..

[20] Sandhaus R.A., Turino G. (2013). Neutrophil elastase-mediated lung disease. COPD J. Chronic Obstr. Pulm. Dis..

[21] Green B.J., Wiriyachaiporn S., Grainge C., Rogers G.B., Kehagia V., Lau L., Carroll M.P., Bruce K.D., Howarth P.H. (2014). Potentially pathogenic airway bacteria and neutrophilic inflammation in treatment resistant severe asthma. PLoS ONE.

[22] Augustyniak D., Majkowska-Skrobek G., Kraus-Filarska M., Jankowski A. (2003). Effects of *M. catarrhalis* antigens on IFN-γ, IL-4 and IL-13 production by PBMC from children with atopic asthma or recurrent respiratory tract infections. Cent. Eur. J. Immunol..

[23] Bisgaard H., Hermansen N., Buchvald F., Loland L., Halkjaer B., Bønnelykke K., Brasholt M., Heltberg A., Vissing H., Thorsen V. (2007). Childhood asthma after bacterial colonization of the airway in neonates. N. Engl. J. Med..

[24] Manolov T., Thuan T.T., Forsgren A., Riesbeck K. (2008). *Moraxella*-dependent α1-antichymotrypsin neutralization: A unique virulence mechanism. Am. J. Respir. Cell Mol. Biol..

[25] Alnahas S., Hagner S., Raifer H., Kilic A., Gasteiger G., Mutters R., Hellhund A., Prinz I., Pinkenburg O., Visekruna A. (2017). IL-17 and tnf-α are key mediators of *Moraxella catarrhalis* triggered exacerbation of allergic airway inflammation. Front. Immunol..

[26] Angrill J., Agustí C., De Celis R., Filella X., Rañó A., Elena M., De La Bellacasa J.P., Xaubet A., Torres A. (2001). Bronchial inflammation and colonization in patients with clinically stable bronchiectasis. Am. J. Respir. Crit. Care Med..

[27] Thulborn S.J., Mistry V., Brightling C.E., Moffitt K.L., Ribeiro D., Bafadhel M. (2019). Neutrophil elastase as a biomarker for bacterial infection in copd. Respir. Res..

[28] Augustyniak D., Roszkowiak J., Wiśniewska I., Skała J., Gorczyca D., Drulis-Kawa Z. (2018). Neuropeptides SP and CGRP diminish the *Moraxella catarrhalis* outer membrane vesicle- (OMV-) triggered inflammatory response of human A549 epithelial cells and neutrophils. Mediators Inflamm..

[29] Luke N.R., Campagnari A.A. (1999). Construction and characterization of *Moraxella catarrhalis* mutants defective in expression of transferrin receptors. Infect. Immun..

[30] Holm M.M., Vanlerberg S.L., Foley I.M., Sledjeski D.D., Lafontaine E.R. (2004). The *Moraxella catarrhalis* porin-like outer membrane protein CD is an adhesin for human lung cells. Infect. Immun..

[31] Lipski S.L., Akimana C., Timpe J.M., Wooten R.M., Lafontaine E.R. (2007). The *Moraxella catarrhalis* autotransporter McaP is a conserved surface protein that mediates adherence to human epithelial cells through its n-terminal passenger domain. Infect. Immun..

[32] Timpe J.M., Holm M.M., Vanlerberg S.L., Basrur V., Lafontaine E.R. (2003). Identification of a *Moraxella catarrhalis* outer membrane protein exhibiting both adhesin and lipolytic activities. Infect. Immun..

[33] Golkar T., Zielinski M., Berghuis A.M. (2018). Look and outlook on enzyme-mediated macrolide resistance. Front. Microbiol..

[34] Perez A.C., Murphy T.F. (2017). A *Moraxella catarrhalis* vaccine to protect against otitis media and exacerbations of COPD: An update on current progress and challenges. Hum. Vaccines Immunother..

[35] Stockley J.A., Walton G.M., Lord J.M., Sapey E. (2013). Aberrant neutrophil functions in stable chronic obstructive pulmonary disease: The neutrophil as an immunotherapeutic target. Int. Immunopharmacol..

[36] Dicker A.J., Crichton M.L., Pumphrey E.G., Cassidy A.J., Suarez-Cuartin G., Sibila O., Furrie E., Fong C.J., Ibrahim W., Brady G. (2018). Neutrophil extracellular traps are associated with disease severity and microbiota diversity in patients with chronic obstructive pulmonary disease. J. Allergy Clin. Immunol..

[37] Block H., Zarbock A. (2021). A fragile balance: Does neutrophil extracellular trap formation drive pulmonary disease progression?. Cells.

[38] Mosca T., Menezes M.C., Silva A.V., Stirbulov R., Forte W.C. (2015). Chemotactic and phagocytic activity of blood neutrophils in allergic asthma. Immunol. Investig..

[39] Nicchi S., Giusti F., Carello S., Utrio Lanfaloni S., Tavarini S., Frigimelica E., Ferlenghi I., Rossi Paccani S., Merola M., Delany I. (2022). *Moraxella catarrhalis* evades neutrophil oxidative stress responses providing a safer niche for nontypeable *Haemophilus influenzae*. iScience.

[40] Murphy T.F., Kirkham C., Liu D.F., Sethi S. (2003). Human immune response to outer membrane protein cd of *Moraxella catarrhalis* in adults with chronic obstructive pulmonary disease. Infect. Immun..

[41] Murphy T.F., Brauer A.L., Aebi C., Sethi S. (2005). Identification of surface antigens of *Moraxella catarrhalis* as targets of human serum antibody responses in chronic obstructive pulmonary disease. Infect. Immun..

[42] Augustyniak D., Mleczko J., Gutowicz J. (2010). The immunogenicity of the liposome-associated outer membrane proteins (OMPs) of *Moraxella catarrhalis*. Cell. Mol. Biol. Lett..

[43] Augustyniak D., Seredyński R., McClean S., Roszkowiak J., Roszniowski B., Smith D.L., Drulis-Kawa Z., MacKiewicz P. (2018). Virulence factors of *Moraxella catarrhalis* outer membrane vesicles are major targets for cross-reactive antibodies and have adapted during evolution. Sci. Rep..

[44] Hare K.M., Seib K.L., Chang A.B., Harris T.M., Spargo J.C., Smith-Vaughan H.C. (2019). Antimicrobial susceptibility and impact of macrolide antibiotics on *Moraxella catarrhalis* in the upper and lower airways of children with chronic endobronchial suppuration. J. Med. Microbiol..

[45] Liu Y.-l., Ding R., Jia X.-m., Huang J.-j., Yu S., Chan H.T., Li W., Mao L.-l., Zhang L., Zhang X.-y. (2022). Correlation of *Moraxella catarrhalis* macrolide susceptibility with the ability to adhere and invade human respiratory epithelial cells. Emerg. Microbes Infect..

[46] Ozuna H., Uriarte S.M., Demuth D.R. (2021). The hunger games: *Aggregatibacter actinomycetemcomitans* exploits human neutrophils as an epinephrine source for survival. Front. Immunol..

[47] Eichelberger K.R., Goldman W.E. (2020). Manipulating neutrophil degranulation as a bacterial virulence strategy. PLoS Pathog..

[48] Eilers B., Mayer-Scholl A., Walker T., Tang C., Weinrauch Y., Zychlinsky A. (2010). Neutrophil antimicrobial proteins enhance *Shigella flexneri* adhesion and invasion. Cell. Microbiol..

[49] du Teil Espina M., Fu Y., van der Horst D., Hirschfeld C., López-Álvarez M., Mulder L.M., Gscheider C., Haider Rubio A., Huitema M., Becher D. (2022). Coating and corruption of human neutrophils by bacterial outer membrane vesicles. Microbiol. Spectr..

[50] Pandey K.C., De S., Mishra P.K. (2017). Role of proteases in chronic obstructive pulmonary disease. Front. Pharmacol..

[51] Standish A.J., Weiser J.N. (2009). Human neutrophils kill *Streptococcus pneumoniae* via serine proteases. J. Immunol..

[52] Park H.J., Kang H.K., Park E., Kim M.K., Park Y. (2022). Bactericidal activities and action mechanism of the novel antimicrobial peptide Hylin a1 and its analog peptides against *Acinetobacter baumannii* infection. Eur. J. Pharm. Sci..

[53] Altschul S.F., Madden T.L., Schäffer A.A., Zhang J., Zhang Z., Miller W., Lipman D.J. (1997). Gapped BLAST and PSI-BLAST: A new generation of protein database search programsitle. Nucleic Acids Res..

[54] Saito R., Matsuoka S., Fujinami Y., Nonaka S., Ichinose S., Kubota T., Okamura N. (2013). Role of *Moraxella catarrhalis* outer membrane protein CD in bacterial cell morphology and autoaggregation. Res. Microbiol..

