# Supplementary material for: The Direct Anti-Virulence but Not Bactericidal Activity of Human Neutrophil Elastase against Moraxella catarrhalis
Source: Int J Mol Sci. 2023 Apr 1;24(7):6607. doi: 10.3390/ijms24076607 (PMC10094786; doi:10.3390/ijms24076607)
Supplement: Supplementary file 1 [file ijms-24-06607-s001.zip › ijms-2244588-supplementary.pdf]

Supplementary Figure S1

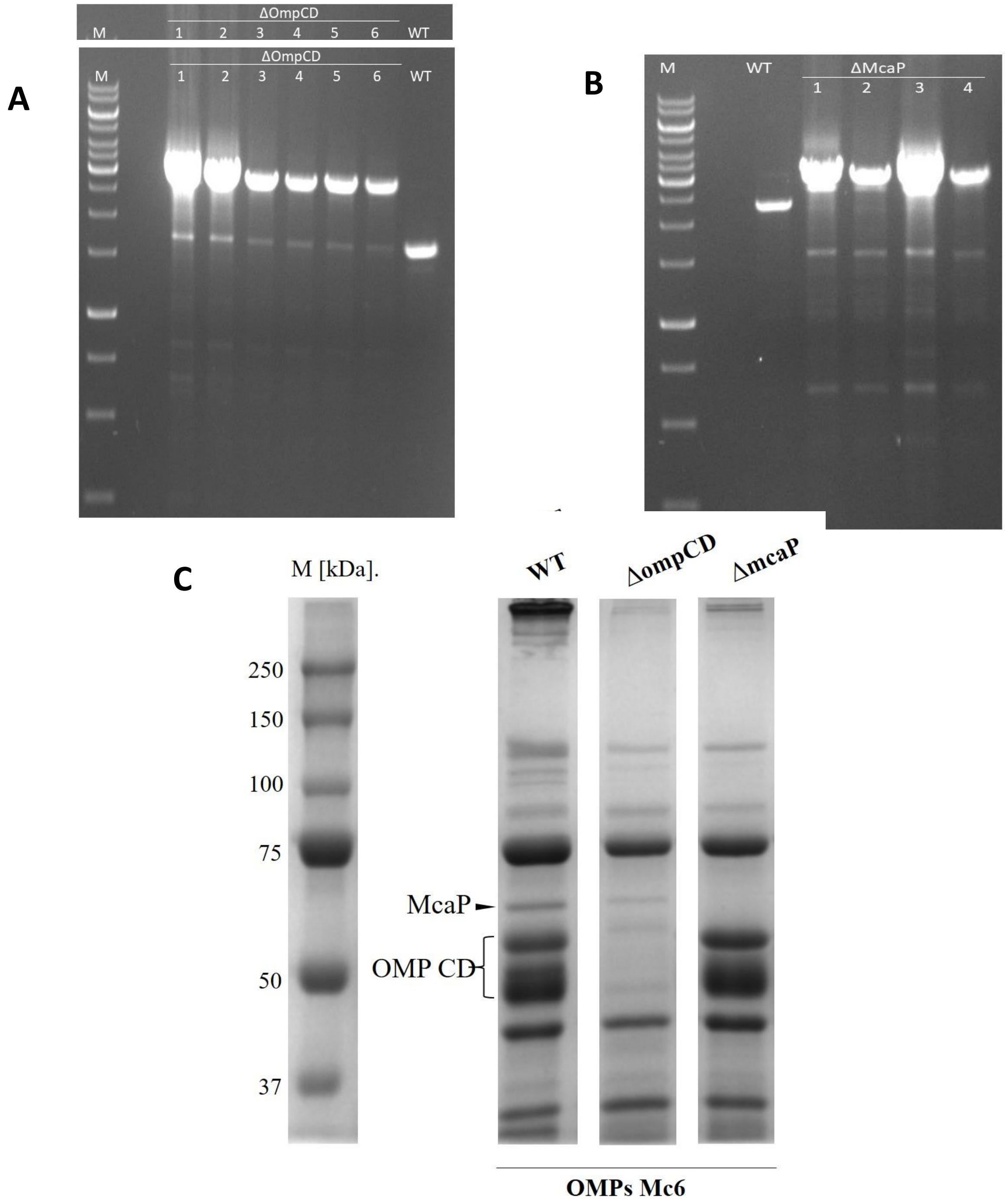

Supplementary Figure S1. Verification of (A) ompCD deletion in *M. catarrhalis 6 ΔompCD* using the primers ompCD-F2 (5’-CATATGGGTGTGACAGTCAGCCCACTAC-3’) and ompCD-R2 (5’-GGATCCCTGGCGATATGCCCGAACTG-3’) as described previously, and (B) mcaP deletion in Δ*mcaP* using the primers P1 (5’-CGCAATAAAGATCACCATGCTTG-3’) and P2 (5’-CGGGATCCCGCTGACACATTGCATTGATAAA-3’) as described before. M- GeneRuler 1 kb DNA Ladder Thermo Scientific; 1-6 clones of Δ*ompCD* Mc6, 1-4 clones of Δ*mcaP* Mc6 WT- wild type of Mc6.

(C) Electrophoretic separation of isolated outer membrane proteins (OMPs) of *M. catarrhalis* Mc6 WT and its isogenic mutants. OMPs were isolated using Zwittergent 3-14 detergent. A Coomassie blue staining of 8% SDS-PAGE gel is shown. The molecular mass standards are indicated on the left.

Supplementary Figure S2

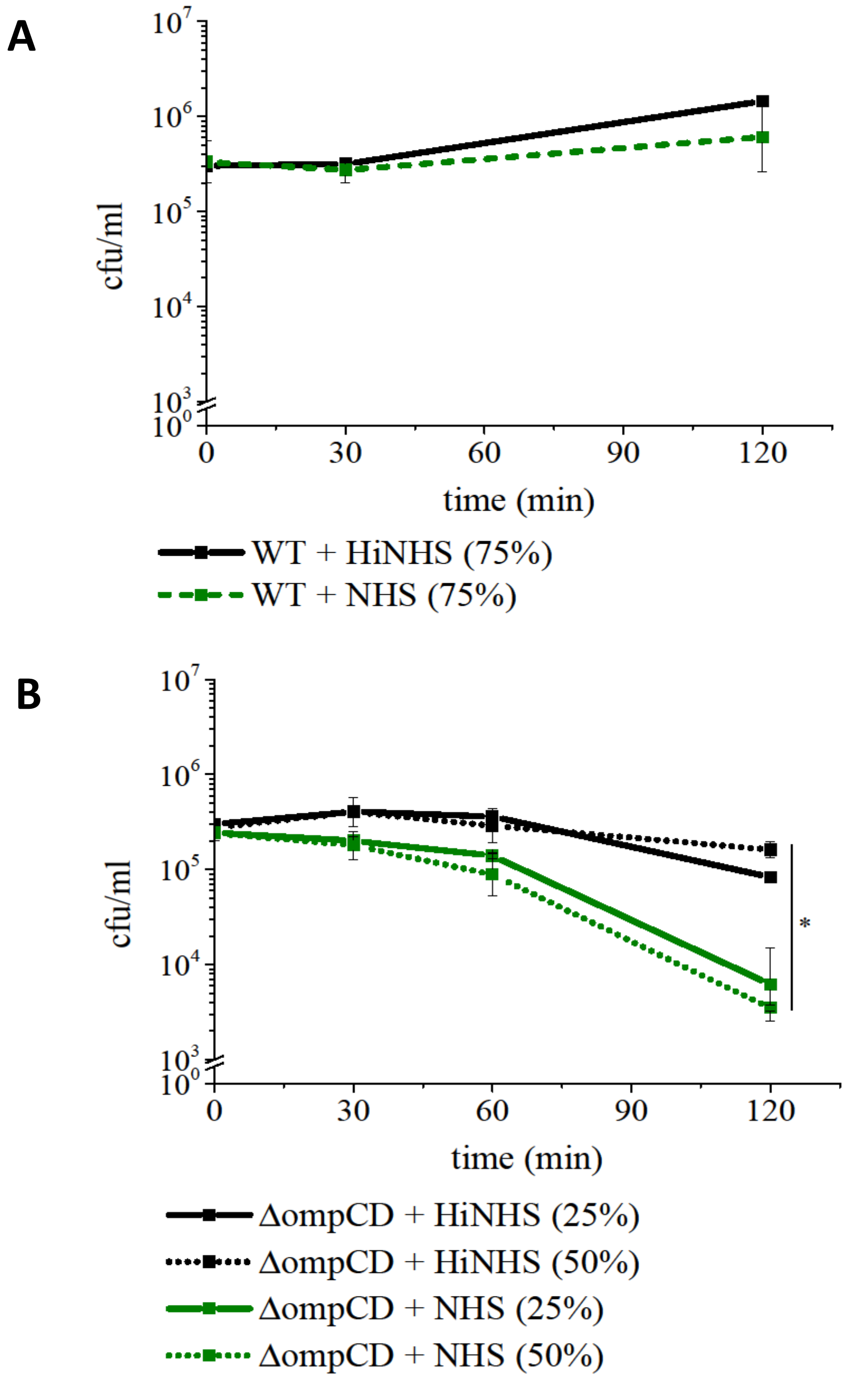

Supplementary Figure S2. Survival of *M.catarrhalis* Mc6 (A) wild-type (WT) strain and (B) isogenic Δ*ompCD* mutant strain in the presence of normal human serum (NHS) complement. As a negative control bacteria treated with heat-inactivated NHS were used (HiNHS). Results are expressed as means cfu/ml ± SD from at least two independent experiments in triplicate. * $p < 0.05$

Supplementary Figure S3

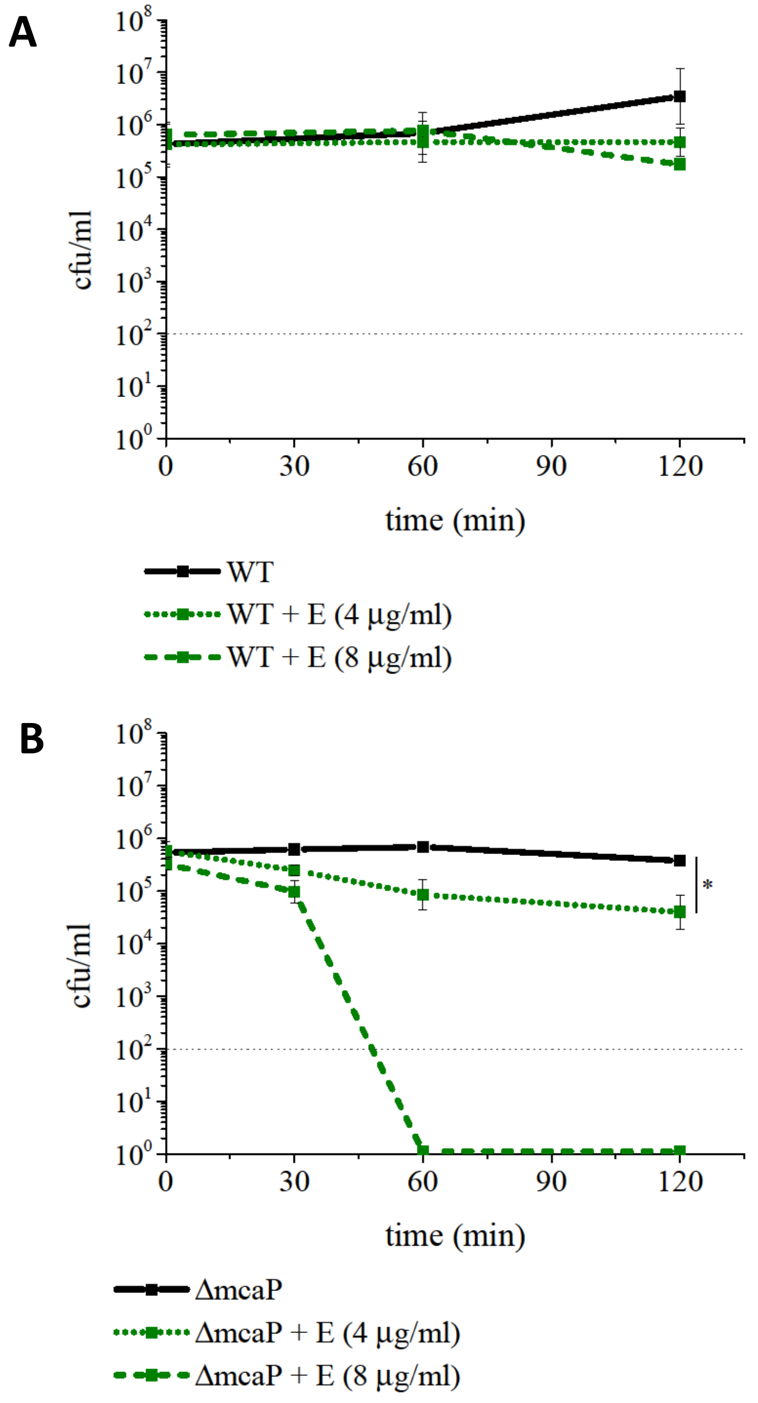

Supplementary Figure S3. Survival of *M.catarrhalis* Mc6 (A) wild-type (WT) strain and (B) isogenic Δ*mcaP* mutant strain in the presence of suprainhibitory concentrations of erythromycin (E). As a negative control bacteria not treated with antibiotic were used. The dashed line indicates the limit of sensitivity of the test (<100 cfu/ml). Results are expressed as means cfu/ml ± SD from at least two independent experiments in triplicate. * $p < 0.05$
